# Setting health sector priorities: a brief overview of Ethiopia’s experience

**DOI:** 10.1186/s12962-018-0117-8

**Published:** 2018-11-09

**Authors:** Mahlet Kifle Habtemariam, Sentayehu Tsegaye Semegn

**Affiliations:** 1 Federal Ministry of Health, Addis Ababa, Ethiopia; 2 Harvard T.H. Chan School of Public Health, 665 Huntington Avenue, Boston, MA 02115 USA; 3 KNCV Tuberculosis Foundation, Addis Ababa, Ethiopia

**Keywords:** Priority setting, Multi-criteria decision analysis (MCDA), Ethiopia

## Abstract

As a country with significant resource constraints, a fair and efficient health priority setting should be at the cornerstone of Ethiopia’s commitment to attain universal health coverage by 2035. This paper draws on the current national strategies including the national essential health service package to explore the criteria and processes used to set the existing national health sector priorities. Additionally, it reviews Ethiopia’s experience in comparison with the multi-criteria decision analysis proposed by Baltussen et al. Finally, the paper highlights the importance of strengthening country-led efforts and investing in human capital to shape priority setting in a developing country context.

## Background

Having a healthy and productive community is a necessity, not a choice, for populous countries like Ethiopia and population can be an asset or a liability depending on the national priorities of the country. With a population of more than 94 million, Ethiopia is the second most populous country in Africa. Close to half of the total population is below the age of 18 years [1]. According to the World Bank’s latest edition of Global Economic Prospects, Ethiopia is one of the fastest growing economies on the globe projected to grow by 8.3% in 2017 [2]. The health care governance as well as its delivery are decentralised and follow a three-tier system. Through the MDG era, creating access to basic primary health care services and building community ownership have been the main priorities of the health sector that resulted in a significant improvement of the population health status and the achievement of most of the health related MDG’s [3]. However, communicable diseases, maternal, childhood and nutritional conditions continue to be rampant, and further compounded by the additional burden of neglected tropical diseases, non-communicable diseases, injuries, and public health emergencies. Quality and equity in the health care delivery remain to pose significant challenges. The per capita health spending is still low, at 28.65 USD, with more than one-third of the total spending coming from development assistance and another third of the spending coming from out of pocket expenditure [4]. Given the existing resource constraints, it is very critical to give priority setting an utmost attention to achieve the sustainable development goals and realise Ethiopia’s vision towards seeing healthy, productive and prosperous citizens.

Two aspects emerge frequently as critical in priority setting for health; the process by which and the criteria used to set health priorities with further elaborations and guidance on both. Clearly defined and agreed upon criteria as well as inclusive and formal processes are equally pertinent for an effective priority setting [5, 6]. Existing literature recommends employment of a systematic process and using multi-criteria decision analysis (MCDA) to help decision makers be objective, and make priority setting more transparent with consistent value trade-offs [6]. Maximizing general population health, distribution of health, specific societal preferences and budgetary and practical constraints were mentioned as some of the criteria by Baltussen et al. [5] in their published work. This paper briefly discusses Ethiopia’s experience in setting health sector priorities at the national level.

Attaining universal health coverage (UHC) by 2035 is the direction for Ethiopia’s health sector development through guaranteeing access to all the essential services, for everyone in need, while providing protection against financial risk [7]. Decisions for priority setting are made at the national, regional, district, and service delivery levels. At the macro level, Ethiopia has a health policy which emphasises on health care decentralisation and prioritisation of health promotion, diseases prevention and basic curative services. At the meso level, strategic documents such as the 20-year envisioning document and the Health Sector Transformation Plan (HSTP) were developed to guide the priorities within the health sector. At the micro level, the Essential Health Service Package (EHSP) has been used as a means to guide service provision with a clear stratification of service delivery and financial arrangements. Exempted services are services that are provided at no charge to all on account of addressing the public health goals. Most of these exempted services fall in the health extension program package. EHSP outside the exempted services are the portions of the EHSP services that a health facility is expected to provide at a minimum cost. These services are heavily subsidised by the government and are provided to individuals who need them on a cost-sharing basis. High cost services are services outside the EHSP domain, which are provided on a high cost recovery basis. In order to address equity, the concept of “fee waiver” was introduced, which is a right conferred to an individual that entitles him or her to obtain health services at no direct charge or at a reduced-price based on the inability to pay. Through the fee waiver system the poor get free access to both the EHSP and the high cost services [8].

Looking at the mechanism for priority setting during the development of the above policy documents, overall, there is a lack of adequate documentation either on the criteria or the processes used. Cost effectiveness, affordability, equity, necessity, capacity and accessibility were the principles/criteria used to select the health service priorities in the EHSP [4]. The EHSP, therefore, considered multiple criteria that resulted in a rank ordering of the different health services. It gave major emphasis to equitable health care access by exempting the basic health care services at the primary health care level and through a fee waiver arrangement for those unable to pay, which is well aligned with the concept of a fair priority setting [9]. On the other hand, the reasons for how the above criteria were selected, weighted and vetted to result in the rank ordering/classification of interventions in the EHSP were not mentioned in the document. Moreover, the criteria were not adequately defined and some of the criteria seem to overlap with each other (for instance cost effectiveness vs. affordability). The Envisioning Ethiopia’s Path towards Universal Health Coverage through Strengthening Primary Health Care (2015–2035) outlines that health care package selection should follow the principles of; (1) protecting the community from adverse consequences of paying for health care, (2) block coverage of priority package of services, (3) selection of the priority package of services based on disease burden and impact, and (4) ensuring quality standards of priority services. The HSTP, which is the first phase of the Envisioning Ethiopia’s Health: 2035, followed similar principles [8, 9].

There is still no clear consensus in the health priority setting literature neither on the set of criteria that should be used nor the weights that should be given. However, a participatory and transparent process for setting priorities stands out as the best approach to ensure a fair priority setting. Ethiopia has a creditable experience in a participatory process for priority setting. Health care planning follows the “One plan, One budget and One report” approach. Both the Envisioning and the HSTP were developed by engaging the relevant stakeholders and guided by a clear roadmap, with the aim of generating commitment and shared vision towards the realisation of the plans and their effective implementation [3]. A Joint Assessment of National Strategies (JANS) was also conducted to ensure alignment of different disease specific health strategies and plans [3]. As Ethiopia’s health sector is significantly dependent on external development assistance, adequate engagement of the donor community is mandatory to guide investment into the priority health care services. Apart from ensuring fairness, a participatory and transparent process during the development of national strategies was shown to meaningfully and significantly influence practice during implementation. The SDG Performance Fund (a mechanism by which available funding from donors is combined and managed by the government) is a good example, among many, where development partners have aligned their priorities towards an overarching agreed upon national health plan. Again, whether the stakeholders engagement was all-inclusive and meaningful needs to be further evaluated and documented.

Although the achievement of major internationally agreed development goals, such as MDGs, can serve as a proxy to indicate that the country is on the right path towards an effective priority setting to address major health challenges of the poor and the most disadvantaged, it does not override the need to put in place an explicit mechanism to set health sector priorities and a means to hold decision makers accountable. The Ministry of Health (MoH) has therefore embarked on different and new initiatives to improve the existing priority setting practice in the health sector. Current efforts include the establishment of an economic analysis unit within the MoH (in 2016), which is responsible to generate context specific evidence on cost effective interventions, annual resource mapping, national health accounts, and other initiatives in relation to effective and efficient use of resources. There are additional efforts such as the Disease Control Priorities-Ethiopia, which was also designed to build the country’s immediate and long term human resource capacity in health economics and economic evaluations as inadequate technical capacity is one of the major bottlenecks.

## Conclusion

Looking forward, we realize a need to strengthen the above country-led efforts and advance the national health priority setting process towards a systematic and rational approach. Dialogue on the existing practice and future plans, improved documentation and generating grounded empirical evidence will help us to better understand our gaps and assets. Improved human resource capacity, context specific evidence, consented criteria, coupled with enhanced process of engaging all the stakeholders will help the country to put in place a systematic priority setting for health.
